# Farm‐Level Risk Factors Associated With the Colonization of Chicken Flocks With *Campylobacter* spp. in Murang′a County, Kenya

**DOI:** 10.1155/ijm/6404666

**Published:** 2026-10-22

**Authors:** Luciano A. Achieng, Daniel W. Wanja, Christine M. Mbindyo, Shepelo Getrude Peter, Felix M. Kibegwa, Mathew Kiplimo, George C. Gitao, Charles D. Kato

**Affiliations:** ^1^ Department of Veterinary Pathology Microbiology and Parasitology Faculty of Veterinary Medicine University of Nairobi Nairobi Kenya uonbi.ac.ke; ^2^ Department of Veterinary Pathology Microbiology and Parasitology Faculty of Veterinary Medicine and Surgery Egerton University Njoro Kenya egerton.ac.ke; ^3^ Department of Clinical Studies Faculty of Veterinary Medicine University of Nairobi Nairobi Kenya uonbi.ac.ke; ^4^ Department of Animal Production Faculty of Veterinary Medicine University of Nairobi Nairobi Kenya uonbi.ac.ke; ^5^ Department of Animal Sciences School of Natural Resources and Animal Sciences (SONRAS) Jomo Kenyatta University Nairobi Kenya jkuat.ac.ke; ^6^ School of Biosecurity Biotechnical and Laboratory Sciences College of Veterinary Medicine Animal Resources and Biosecurity Makerere University Kampala Uganda mak.ac.ug

**Keywords:** *Campylobacter* spp., farm biosecurity, poultry health, smallholder poultry systems, zoonotic bacteria

## Abstract

Poultry production is a key source of income and animal protein in Kenya, especially for smallholders. Chickens are important reservoirs of zoonotic bacteria such as *Campylobacter* spp., which can infect humans via direct contact or contaminated products. Identifying farm‐level factors linked to *Campylobacter* colonization in chickens is crucial for improving biosecurity and reducing public health risks. This cross‐sectional study was conducted in four selected subcounties of Murang′a County between August and December 2024 and aimed to identify farm‐level risk factors associated with *Campylobacter* colonization in the chicken gut in Murang′a County, Kenya. We collected 340 cloacal swabs from chickens reared on smallholder farms. Swabs were processed using standard bacteriological isolation and preliminary phenotypic identification, followed by genus‐level confirmation using matrix‐assisted laser desorption/ionization–time of flight mass spectrometry (MALDI‐TOF MS). Epidemiological data on potential risk factors were collected using a pretested semistructured questionnaire administered to poultry farmers. Logistic regression analysis was used to evaluate associations between farm‐level factors and isolation of *Campylobacter* spp. in chickens. Among the 340 farms, 215 were positive for *Campylobacter* spp., resulting in a farm‐level prevalence of 63.2% (95% CI: 58.1%–68.3%). *Campylobacter* colonization was significantly associated with poor poultry house hygiene (OR = 2.880, *p* = 0.018), larger flock size (OR = 2.678, *p* = 0.017) and higher farmer education level (*OR* = 1.984, *p* = 0.044). The education association likely suggests a proxy for production intensity rather than an independent biological risk factor. *Campylobacter* occurrence is influenced by farm hygiene, farmer education and flock size in smallholder poultry systems. Improving farm biosecurity practices, farmer awareness and hygiene could reduce zoonotic bacterial risk along the poultry value chain in Murang′a County and similar smallholder production systems.

## 1. Introduction

Poultry production is vital for food security, nutrition and rural livelihoods in many low‐ and middle‐income countries, including Kenya. Smallholder systems that use indigenous and improved local breeds are common because they require little capital and offer rapid returns. Chickens provide accessible, high‐quality animal protein and income for rural households, supporting both household economies and national agricultural output. However, poultry systems can be major reservoirs for zoonotic pathogens such as *Campylobacter* spp., which are leading causes of foodborne illness globally and in Africa, causing millions of cases annually [1]. Contaminated poultry meat is a primary source of human infection [2], and poultry products are estimated to cause 20%–30% of human campylobacteriosis cases in many regions [3]. Retail chicken meat frequently carries pathogenic *Campylobacter*, with reported prevalence of up to 64% in some markets [4]. Prevalence estimates vary widely due to differences in production systems, farm biosecurity, diagnostic methods and environmental conditions that affect bacterial survival and transmission [5–7].

In Kenya, studies across the poultry value chain report a high prevalence of *Campylobacter* species. Carron et al. [2] reported *Campylobacter* in 33%–44% of small‐scale peri‐urban farms and in 60%–64% of retail chicken meat. Mageto et al. [8] similarly reported high infection rates in broilers, layers and indigenous chickens in peri‐urban systems. Studies in live bird markets in Busia and broiler farms in Thika reported *Campylobacter* colonization rates of 29%–44% in live birds, with carcass contamination at approximately 30% [9, 10]. These studies underscore rapid bacterial colonization and the importance of production and hygiene practices in transmission along the poultry value chain.

Farm‐level practices are key determinants of pathogen persistence and spread in poultry systems. Studies on Kenyan smallholder and peri‐urban poultry farms have shown that poor coop hygiene, weak biosecurity measures, reuse of contaminated litter, absence of footbaths, use of housing that is difficult to clean and disinfect and unrestricted human movement between flocks significantly increase the risk of pathogen introduction and dissemination [2, 8, 9]. These conditions promote environmental contamination and rapid horizontal transmission of enteric bacteria within and between flocks. In addition to farm management, chicken‐level factors such as flock size and stocking density influence bacterial transmission. Larger flocks and higher stocking densities increase bird‐to‐bird contact and environmental contamination, thereby promoting the spread of *Campylobacter* spp. [2, 6, 11, 12]. These findings indicate that management‐related factors, rather than isolated exposure events, are central to maintaining zoonotic bacterial transmission in poultry systems. In Kenya, most studies on *Campylobacter* spp. in poultry emphasize pathogen detection rather than farm‐level determinants of colonization. As a result, empirical evidence linking production practices, biosecurity and management factors to the occurrence of these bacteria in smallholder poultry farms is limited.

Murang′a County in central Kenya is a key poultry‐producing region with high population density, numerous smallholder farms and frequent human–poultry contact [13]. Poultry are raised primarily in free‐range or semi‐intensive systems, with limited veterinary support and inconsistent biosecurity. Combined with informal marketing and environmental conditions that favour bacterial survival, these factors promote the persistence and transmission of zoonotic pathogens throughout the poultry value chain. Understanding farm‐level risk factors for bacterial colonization in poultry is essential for designing targeted interventions to improve biosecurity and reduce zoonotic exposure. This study therefore aimed to identify farm‐level risk factors for *Campylobacter* spp. colonization in chickens from smallholder poultry farms in Murang′a County, Kenya. These findings are expected to support improved poultry health management, increase food safety and reduce public health risks from zoonotic bacteria in smallholder poultry systems.

## 2. Materials and Methods

### 2.1. Ethical Statement

The study protocol and procedures were reviewed and approved by the Biosafety Animal Use and Ethical Committee, Faculty of Veterinary Medicine, University of Nairobi (Reference No. FVM BAUEC/2024/579). Informed written consent to conduct the study in Murang′a County was obtained from the County Director of Veterinary Services (CDVS). Verbal consent was then obtained from poultry farm owners/operators before data collection at sampling, after which the authors explained the study objectives and data to be collected.

### 2.2. Study Areas and Sites

This study was conducted in Murang′a County in central Kenya, approximately 84 km north of Nairobi (Figure 1). The county borders Kiambu to the south, Nyeri to the north, Nyandarua to the west and Machakos, Embu and Kirinyaga to the east. It lies between 0°43 ^′^0 ^″^S and 1°10 ^′^0 ^″^S and between 36°48 ^′^0 ^″^E and 37°10 ^′^0 ^″^E. Administratively, Murang′a has nine subcounties: Gatang′a, Ithang′a, Kahuro, Kandara, Kangema, Kigumo, Mathioya, Murang′a East and Murang′a South. According to KNBS (2019), it contributes 2.3% of Kenya′s GDP, with agriculture as the main economic activity. It is one of Kenya′s leading rural counties in terms of agricultural production, dominated by smallholder mixed farming and high livestock densities.

**Figure 1:**
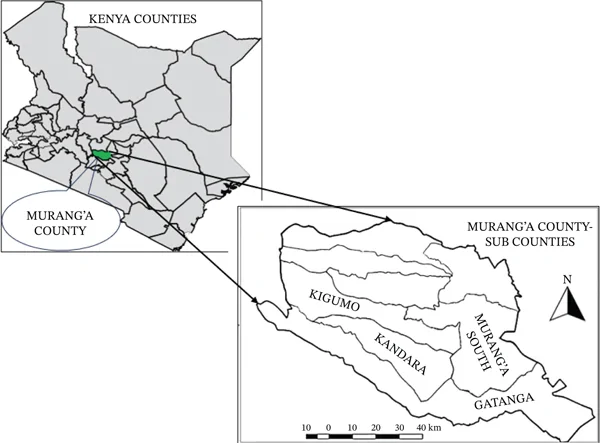
Map of Kenya showing the location of Murang′a county and the selected subcounties included in the study.

The study sites were Kandara, Murang′a South, Gatang′a and Kigumo subcounties, which were purposively selected as high‐potential agricultural areas with dense populations and substantial poultry production. They are characterized by small landholdings and good accessibility, intensifying human–animal interactions and facilitating poultry marketing due to their proximity to Nairobi. Poultry production in these subcounties is mostly small‐scale and integrated into mixed farming systems, making them suitable for investigating foodborne bacteria in high‐density smallholder poultry systems.

### 2.3. Study Design

A cross‐sectional survey of 340 chicken farms was conducted from August to December 2024 in Murang′a County. Cloacal swabs were collected from each farm via standard veterinary and microbiological protocols. Bacterial isolation and preliminary identification were performed using standard bacteriological methods and confirmed via Matrix‐Assisted Laser Desorption/Ionization Time‐of‐Flight Mass Spectrometry (MALDI‐TOF MS). A pre‐tested, semistructured questionnaire was administered in all farms to assess potential risk factors associated with the occurrence of *Campylobacter* spp.

### 2.4. Study Population

The target population for this study comprised households involved in small‐scale poultry production, specifically those rearing indigenous chickens, improved or mixed breeds, broilers or layers, and managing these flocks under intensive, semi‐intensive, or free‐range production systems.

### 2.5. Sample Size Determination

The sample size was calculated via the formula *n* = (*Z* ^2^ *P*(1 − *P*))/*d* ^2^ [14], where *n* is the minimum required sample size, *Z* is the standard normal deviation for a 95% confidence level (1.96), *d* is the desired precision (5%; 0.05) and *P* is the anticipated farm‐level prevalence of *Campylobacter* spp., set at 33% on the basis of previous studies [2]. This yielded a final sample size of 340 farms across the study area. The number of farms sampled per subcounty varied according to poultry farm availability and farm owners′ or operators′ willingness to participate at the time of data collection.

### 2.6. Eligibility Criteria for Participating in Farms

This study included small‐scale poultry farms (households) keeping between 10 and 100 birds, comprising local chickens, improved or mixed breeds, broilers, or layers, managed under intensive, semi‐intensive, or free‐range systems. The study excluded homesteads not engaged in poultry keeping and those that declined to participate.

### 2.7. Data Collection Through Researcher Observations and Questionnaire Administration

Before data collection, the study objectives and sampling procedures were explained, and written informed consent was obtained; only consenting farms were included. Epidemiological data were collected through face‐to‐face interviews and systematic on‐site observations using a pretested semistructured questionnaire. Formal structured interviews were administered via a Google Forms questionnaire. The survey recorded demographic data (gender, age and education), farm characteristics (flock size, breed type and other animals), poultry management practices (housing, feeding and manure disposal) and existing farm biosecurity measures. In addition to the questionnaire interview, the researcher assessed poultry house hygiene through direct observation during farm visits to evaluate its potential association with *Campylobacter* presence. Two observable indicators—cleanliness and wetness of the poultry house/enclosure—were assessed and recorded using predefined categories in the data collection tool. The same criteria were applied consistently across all participating farms to ensure uniformity of assessment. On‐site observations minimized recall bias and verified information from 340 farm owners or poultry workers. All data were anonymized to maintain confidentiality and protect participants′ privacy.

### 2.8. Sample Collection, Laboratory Isolation and Identification of *Campylobacter* spp.

Following standard procedures described by OIE [15] and USDA‐FSIS [16], cloacal swabs were aseptically collected from five birds per farm using sterile cotton swabs and pooled to form a single composite sample for each farm. Bacteriological analyses were performed at the Central Veterinary Laboratories (CVL), Kabete, using conventional culture and isolation techniques following standard bacteriological methods [17]. Due to the approximately 4‐h interval between sample collection in Murang′a County and laboratory processing in Nairobi, samples were subjected to selective enrichment using Preston broth prior to isolation of *Campylobacter* spp. The use of enrichment was intended to support recovery of potentially stressed organisms following sample transport. Composite samples were selectively enriched in Preston broth for the isolation of *Campylobacter* spp., followed by streaking on Modified Preston Campylobacter Agar Base (Oxoid) supplemented with selective additives. A limitation of this approach was that enrichment and direct plating were not performed in parallel. Therefore, the relative recovery efficiency of the two approaches could not be assessed, and the extent to which competitive inhibition by background flora may have affected *Campylobacter* recovery could not be quantified.

Presumptive *Campylobacter* isolates were identified to the genus level on the basis of characteristic colony morphology (small, round, grey to translucent, moist and slightly raised colonies), Gram staining reactions [slender Gram‐negative, curved or spiral (‘gull‐wing’) rods] and positive catalase and oxidase reactions. All presumptive *Campylobacter* isolates were identified and confirmed to belong to the genus *Campylobacter* using matrix‐assisted laser desorption/ionization–time of flight mass spectrometry (MALDI‐TOF MS; Bruker Daltonics GmbH, Bremen, Germany). Identification followed standard Bruker scoring criteria: Scores ≥ 2.0 indicated reliable species‐level identification, scores ≥ 1.7 to < 2.0 indicated genus‐level identification and scores < 1.7 were considered unreliable [18].

A flock was considered positive if *Campylobacter* spp. were detected; otherwise, it was classified as negative.

### 2.9. Data Management and Analysis

All the data collected were initially entered into Microsoft Excel (2016) and subsequently analysed via the Statistical Package for the Social Sciences (SPSS) Version 26. Preliminary data exploration involved descriptive statistics to summarize variable distributions and calculate pathogen prevalence rates, with prevalence determined as the proportion of positive samples relative to the total number of samples tested. To assess associations between potential risk factors and bacterial presence, odds ratios (ORs) were estimated using logistic regression analyses. Variables with *p* < 0.25 in the univariable logistic regression were selected for inclusion in the multivariable logistic regression model, where *p* < 0.05 was considered statistically significant.

## 3. Results

### 3.1. Characteristics of the Farms Surveyed

Table 1 shows the distribution of farm characteristics among the 340 poultry farms surveyed across the four subcounties. Among the 340 farms, Murang′a South had the largest share (29.4%, 95% CI: 24.8–34.5). Most farms were managed by women (52.4%, 95% CI: 47.0%–57.6%). Semi‐intensive systems were most common (40.6%, 95% CI: 35.5–45.9), followed by free‐range systems (35.9%, 95% CI: 30.9–41.1). Most farmers kept local (Kienyeji) breeds (66.5%, 95% CI: 61.3–71.3), typically with 11–50 birds (62.1%, 95% CI: 56.8–67.1). Tap water was the main poultry water source (42.9%, 95% CI: 37.8%–48.3%).

**Table 1:** Farm characteristics of surveyed poultry farms (*n* = 340) with proportions and 95% confidence intervals.

Variable	Category	*N*	Percentage (%)	95% CI
Number of farms surveyed per subcounty	Murang′a South	100	29.4	24.8–34.5
	Kigumo	83	24.4	20.1–29.2
	Gatanga	81	23.8	19.6–28.6
	Kandara	76	22.4	18.2–27.1

Gender of farm owner/caretaker	Female	178	52.4	47.0–57.6
	Male	162	47.6	42.4–53.0

Poultry production system	Semi‐intensive	138	40.6	35.5–45.9
	Free‐range	122	35.9	30.9–41.1
	Intensive	39	11.5	8.5–15.3
	Backyard	17	5.0	3.1–7.9

Chicken breed kept	Local (Kienyeji)	226	66.5	61.3–71.3
	Improved Kienyeji	80	23.5	19.3–28.3
	Layers	23	6.8	4.5–9.9
	Broilers	10	2.9	1.6–5.3

Flock size	≤ 10 birds	81	23.8	19.6–28.6
	11–50 birds	211	62.1	56.8–67.1
	51–100 birds	24	7.1	4.8–10.3
	> 100 birds	24	7.1	4.8–10.3

Water source for poultry	Tap water	146	42.9	37.8–48.3
	Borehole	68	20.0	16.1–24.6
	River	26	7.6	5.3–10.9
	Well	16	4.7	2.9–7.5

*Note:* 95% CI = 95% confidence interval (CI) calculated using the binomial exact method.

### 3.2. Farm‐Level Prevalence of Thermophilic *Campylobacter* Species in Chicken Flocks

*Campylobacter*‐like colonies were found in 215 of 340 samples. Biochemical tests revealed that all 215 isolates were Gram‐negative, catalase‐positive and oxidase‐positive curved rods. Matrix‐assisted laser desorption/ionization‐TOF confirmed all 215 to be *Campylobacter* spp., giving a farm‐level prevalence of 63.2% (215/340).

### 3.3. Analysis of Independent Variables for the Occurrence of Campylobacter Species on the Study Farms in Murang′a County

*Campylobacter* spp. occurrence varied according to flock characteristics, management and hygiene factors (Table 2). Layer flocks had the highest positivity (82.6%). Farms that fed chickens on both commercial feed and kitchen waste had 82.4% positivity. A total of 76.2% of the flocks on farms with disinfected footbaths were positive, and 71.8% of those on intensive systems were positive. More frequent cleaning was sometimes linked to higher detection: farms cleaning houses twice weekly and weekly had positivity rates of 90.0% and 69.6%, respectively. Larger flocks (≥ 101 birds) also presented higher detection rates (70.8%).

**Table 2:** Descriptive summary of independent variables for the occurrence of *Campylobacter* species on the study farms (*n* = 340).

Variable	Category	Total (n)	*Campylobacter* positivity *n* (%)
Chicken breed	Kienyeji/local	226	137 (60.6)
	Mixed breeds/improved Kienyeji	80	51 (63.7)
	Layers	23	19 (82.6)
	Broilers	10	6 (60.0)
	Other	1	1 (100.0)

Drinker cleaning frequency	Once daily	234	149 (63.7)
	Once a week	63	37 (58.7)
	Once a month	12	4 (33.3)
	Never	2	1 (50.0)
	Other frequencies	29	23 (79.3)

Wetness and cleanliness of enclosure	Dry	146	91 (62.3)
	Moderately dry	122	80 (65.6)
	Very dry	49	32 (65.3)
	Wet and dirty	17	10 (58.8)
	Very wet, dirty and slippery	6	1 (16.7)

Type of feed	Purchased/commercial	183	117 (63.9)
	Purchased/commercial, cereals, kitchen waste	50	30 (60.0)
	Purchased/commercial, cereals	21	13 (61.9)
	Purchased/commercial, kitchen waste	17	14 (82.4)
	Cereals	14	7 (50.0)
	Other feed combinations	55	33 (60.0)

Footbath with disinfectant	No	319	198 (62.1)
	Yes	21	16 (76.2)

Hand cleaning after handling chicken	No	150	95 (63.3)
	Yes	189	119 (63.0)
	Other	1	0 (0.0%)

Place of slaughter	At home	159	97 (61.0)
	N/A (various responses)	89	57 (64.0)
	Sells alive/live birds	63	42 (66.7)
	At the market	9	6 (66.7)
	Slaughterhouse	5	4 (80.0)
	Other locations/responses	15	8 (53.3)

Product transport method	N/A (various responses)	95	54 (56.8)
	In a bag	80	49 (61.3)
	In a box	35	25 (71.4)
	Live bird sales	50	31 (62.0)
	Other transport methods	80	55 (68.8)

Education level	No formal education	8	5 (62.5)
	Primary level	101	64 (63.4)
	Secondary level	155	99 (63.9)
	Tertiary level	76	46 (60.5)

Chicken house cleaning frequency	Weekly	138	96 (69.6)
	Daily	84	53 (63.1)
	Monthly	70	31 (44.3)
	After every production cycle	10	5 (50.0)
	Twice a week	10	9 (90.0)
	Never	4	3 (75.0)
	Other frequencies	24	17 (70.8)

Age separation of chickens	No (ages separated)	165	109 (66.1)
	Yes (all ages together)	175	105 (60.0)

Flock size	≤ 10	81	52 (64.2)
	11–50	211	129 (61.1)
	51–100	24	16 (66.7)
	≥ 101	24	17 (70.8)

Production type	Semi‐intensive (backyard production)	138	82 (59.4)
	Free range	122	82 (67.2)
	Intensive production	39	28 (71.8)
	Free range	24	14 (58.3)
	Backyard production	17	8 (47.1)

Vaccination schedule adherence	No	235	145 (61.7)
	Yes	105	69 (65.7)

Visitor access to shed	No	208	129 (62.0)
	Yes	132	85 (64.4)

### 3.4. Univariate and Multivariate Analyses of Potential Risk Factors for the Occurrence of *Campylobacter* Species

Ten potential risk factors were evaluated via univariable logistic regression (Table 3). At the univariable level, three factors are linked to *Campylobacter* occurrence: larger flock size, higher farmer education and poor poultry house hygiene. The odds of detection were greater in flocks of ≥ 101 birds than in those of ≤ 10 birds (OR 2.12; *p* = 0.025), farms with secondary/tertiary‐educated farmers (OR 1.42; *p* = 0.023) and farms where hygiene was poor (wet and/or dirty) (OR 2.88; *p* = 0.018). Bird age separation, production system, breed, vaccination, visitor access, footbath use and handwashing were not significant (*p* > 0.05).

**Table 3:** Univariate logistic regression analysis of potential risk factors associated with *Campylobacter* occurrence.

Variable	Level	OR	95% CI	*p*
Flock size	≤ 10 birds (Ref)	1.000	—	—
	11–50 birds	1.632	0.996–2.674	0.052
	51–100 birds	1.432	0.667–3.075	0.358
	≥ 101 birds	2.117	1.101–4.072	0.025 ^∗^

Education level	Primary/no formal (ref)	1.000	—	—
	Secondary/tertiary	1.420	1.051–1.919	0.023 ^∗^

Poultry house hygiene	Good (dry/clean) (ref)	1.000	—	—
	Poor (wet/dirty)	2.880	1.200–6.910	0.018 ^∗^

Separation of birds by age	Ages together (ref)	1.000	—	—
	Ages separated	0.732	0.472–1.136	0.165

Production system	Semi‐intensive (ref)	1.000	—	—
	Free range	1.388	0.854–2.256	0.187
	Intensive	1.733	0.843–3.562	0.135

Breed type	Kienyeji/local (ref)	1.000	—	—
	Mixed/improved Kienyeji	1.142	0.693–1.884	0.602
	Layers	3.092	0.973–9.824	0.056
	Broilers	0.977	0.275–3.474	0.972

Vaccination schedule	No (ref)	1.000	—	—
	Yes	1.189	0.737–1.917	0.479

Visitors allowed in poultry shed	No (ref)	1.000	—	—
	Yes	1.106	0.706–1.734	0.659

Footbath with disinfectant	No (ref)	1.000	—	—
	Yes	0.964	0.386–2.410	0.936
Handwashing after handling	No (ref)	1.000	—	—
	Yes	1.030	0.671–1.580	0.881

Abbreviations: CI, 95% confidence interval; OR, odds ratio.

∗Significant at 95% CI.

Multivariate logistic regression identified poultry house hygiene, farmer education level and flock size as independent predictors of *Campylobacter* occurrence (Table 4). Farms with poor hygiene (wet and/or dirty poultry house) had 2.9 times greater odds than those with good hygiene (clean/dry poultry house) did (OR = 2.88, 95% CI: 1.20–6.91; *p* = 0.018). Farms managed by farmers with secondary or tertiary education also had higher odds than those with primary or no formal education did (OR = 1.98, 95% CI: 1.02–3.87; *p* = 0.044). Compared with farms with ≤ 10 birds, those with 11–50 birds (OR = 2.12, 95% CI: 1.09–4.12; *p* = 0.027) and ≥ 101 birds (OR = 2.68, 95% CI: 1.19–6.01; *p* = 0.017) had significantly greater odds.

**Table 4:** Multivariable analysis of farm management factors associated with *Campylobacter* occurrence in poultry farms in Murang′a county.

Variable	Level	OR	*p*	95% CI
Poultry house hygiene	Poor hygiene (wet/dirty)	2.880	0.018 ^∗^	1.200–6.910
	Good hygiene (dry/clean)	Reference	—	—

Education level	Secondary/tertiary	1.984	0.044 ^∗^	1.018–3.867
	Primary/no formal education	Reference	—	—

Flock size	≥ 101 birds	2.678	0.017 ^∗^	1.194–6.007
	11–50 birds	2.117	0.027 ^∗^	1.087–4.122
	≤ 10 birds	Reference	—	—

Abbreviations: CI, 95% confidence interval; OR, odds ratio.

∗Significant at 95% CI.

## 4. Discussion

Poultry meat is one of the most affordable and widely consumed animal proteins, especially in low‐ and middle‐income countries, but its safety and quality can be compromised by infectious agents and poor management in production systems [19, 20]. This study underscores the public health importance of *Campylobacter* spp. in smallholder poultry systems in Murang′a County, Kenya. Globally, *Campylobacter jejuni* and *Campylobacter coli* are major causes of bacterial gastroenteritis in humans, with poultry being the main reservoir [21–23].

*Campylobacter* spp. occurrence varied across farms, indicating a stronger influence of environmental and management factors. These results align with findings from Kenya and other African countries, where inadequate sanitation and environmental contamination are key risk factors for *Campylobacter* colonization in smallholder poultry systems [24–26].

Multivariate logistic regression analysis revealed that poor poultry house hygiene (wet and dirty), large flock size and higher farmer education level were significant risk factors associated with *Campylobacter* colonization in chickens. Farms with poor hygiene practices were approximately 2.88 times more likely to have *Campylobacter*‐positive birds than farms maintaining good hygiene standards were. Poor hygiene likely enables pathogen persistence in litter, drinkers, feeders and surfaces, promoting bird‐to‐bird transmission [27, 28]. This finding underscores the importance of effective cleaning and disinfection practices for reducing environmental contamination and interrupting pathogen transmission within poultry houses. Similarly, larger flock sizes were associated with higher *Campylobacter* prevalence, reflecting density‐dependent transmission. Increased bird contact, litter moisture and ammonia in larger flocks may impair immunity and promote spread, as shown in African and international studies [28–30]. Higher farmer education was also linked to greater *Campylobacter* occurrence. The link between higher farmer education and *Campylobacter* occurrence likely reflects farm management differences, not education itself. More educated farmers often run larger commercial systems with higher stocking densities, frequent bird movement and greater human/equipment traffic. These conditions increase opportunities for *Campylobacter* introduction and spread. Thus, education may be a proxy for production intensity rather than a direct biological risk factor. These findings align with those of previous studies that linked inadequate hygiene, high stocking densities and weak biosecurity to increased *Campylobacter* occurrence in poultry systems [3, 28, 31, 32]. Therefore, strengthening farmer training and improving farm‐level biosecurity could play a key role in reducing *Campylobacter* colonization and the associated public health risks from poultry products.

Although univariable screening identified additional variables including production type, breed type, age separation and vaccination status, none of the variables remained significant in the multivariable models, likely because of limited power, residual confounding or their indirect influence on *Campylobacter* colonization. Similarly, prior work suggests that farm‐level risk factors often act cumulatively or indirectly, making modest effects difficult to detect in cross‐sectional studies [6, 33].

These findings have important implications for poultry production in Kenya and other smallholder systems across Africa. As a zoonotic pathogen, *Campylobacter* poses a significant public health risk, especially where biosecurity is weak and poultry products are minimally processed. Improving farm hygiene, regular cleaning and disinfection of poultry houses, proper litter management, controlled access to sheds and targeted farmer training could substantially lower *Campylobacter* prevalence and enhance food safety [24–26, 34]. Overall, this study revealed that *Campylobacter* colonization is largely driven by farm management and biosecurity practices. These represent key intervention points for improving poultry health, food safety and control of zoonotic transmission.

This study has several limitations. First, its cross‐sectional design prevents the establishment of causal links between risk factors and bacterial occurrence. Second, reliance on farm‐level questionnaire data may introduce reporting bias. In addition, the approximately 4‐h delay between sample collection and laboratory processing raised a concern regarding the potential stress of *Campylobacter* cells during transport, and selective enrichment using Preston broth was therefore adopted as the study protocol. However, because enrichment and direct plating were not performed in parallel, the relative recovery efficiency of the two approaches could not be assessed. Consequently, the extent to which competitive inhibition by background flora may have affected *Campylobacter* recovery cannot be quantified. Nonetheless, this study offers important insights into zoonotic bacteria in smallholder poultry systems in Kenya and highlights the need for improved biosecurity, farmer training and surveillance to reduce foodborne pathogen risk. Future work should include molecular characterization of isolates, antimicrobial resistance profiling and longitudinal monitoring of farms to clarify zoonotic pathogen transmission dynamics in poultry systems.

## 5. Conclusions

This study revealed that *Campylobacter* spp. colonization varied across farms and was significantly linked to poultry house hygiene, flock size and farmer education, emphasizing the role of environmental and management factors. These results highlight the need to strengthen farm‐level biosecurity, ensure regular cleaning and sanitation and train farmers in best practices to reduce *Campylobacter* colonization and human exposure. Targeted interventions in Kenyan smallholder poultry systems could improve poultry health and food safety and reduce zoonotic transmission along the poultry value chain.

## Funding

This research was supported by the Consortium for Advanced Research Training in Africa (CARTA), which is jointly led by the African Population and Health Research Center and the University of the Witwatersrand. The CARTA is funded by the Carnegie Corporation of New York (Grants G‐CS‐24‐62008 and G‐PS‐23‐60922), Sida (Grant 16604), the Norwegian Agency for Development Cooperation (Norad; Grant QZA‐21/0162), the Oak Foundation (Grant OFIL‐24‐091) and the Science for Africa Foundation for the Developing Excellence in Leadership, Training and Science in Africa (DELTAS Africa) programme (Del‐22‐006), with support from Wellcome and the UK Foreign, Commonwealth & Development Office, and is part of the EDCPT2 programme supported by the European Union. The statements and views expressed are solely those of the authors.

## Consent

The authors have nothing to report.

## Conflicts of Interest

The authors declare no conflicts of interest.

## Supporting information


**Supporting Information** Additional supporting information can be found online in the Supporting Information section. File S1: Structured questionnaire for assessing farm‐level risk factors associated with *Campylobacter* colonization in the chicken gut in Murang′a County, Kenya. The questionnaire was digitized via Google Forms and administered through face‐to‐face interviews with 340 small‐scale poultry farmers in Murang′a County.

## Data Availability

The data supporting the findings of this study are available from the corresponding author upon reasonable request.
