# Supplementary material for: Farm‐Level Risk Factors Associated With the Colonization of Chicken Flocks With Campylobacter spp. in Murang′a County, Kenya
Source: Int J Microbiol. 2026 Oct 22;2026:6404666. doi: 10.1155/ijm/6404666 (PMC13689539; doi:10.1155/ijm/6404666)
Supplement: Supplementary file 1 [file IJM-2026-6404666-s001.docx]

**Supplementary File S1:** A Questionnaire to Assess Farm-Level Risk Factors Associated with Colonization of *Campylobacter* spp. in Chickens in Murang’a County, Kenya

**UNIVERSITY OF NAIROBI FACULTY OF VETERINARY MEDICINE**

**DEPARTMENT OF VETERINARY PATHOLOGY, MICROBIOLOGY & PARASITOLOGY**

A Questionnaire to assess farm-level risk factors associated with colonization of *Campylobacter* spp. in chickens in Murang’a county, Kenya

Dear farm owner/caretaker,

I **…………………..**, a principal investigator is currently undertaking a study to investigate farm-level risk factors associated with colonization of *Campylobacter* in chicken gut in Murang’a County, Kenya. You are sincerely invited to participate in this survey by filling in the following questionnaire. Your contribution is precious and will be highly appreciated. Information generated from this questionnaire will be kept confidential and only used for academic purposes where all identities are not disclosed. This is to seek your consent and get your help. Thank you!

Consent granted: **Yes/No.**

A. **Farm identification	Date of Interview…….**
   1. Sub County..................................Ward………………………….Village.........................
   2. GPS reading: Eastings……………………….Southings…………………Altitude…….
B. **Farmer’s Bio data**
1. Name of the farm owner/caretaker …………………………Mobile No…………………
2. Age of the farm owner/caretaker

**[1]** 18-24 years	**[2]** 25-31 years	**[3]** 31-36 years	**[4]** 37 years and above

3. Gender of farm owner/caretaker **[1]** Male	**[2]** Female
4. Education level of the farm owner/caretaker

**[1]** No Formal Education	**[2]** Primary Level	**[3]** Secondary Level **[4]** Tertiary Level

5. Level of income farm owner/caretaker?..........................................................................
6. What is your (respondent’s) responsibility at the farm?

**[1]** Owner	[2] Worker	[3] Other (Specify)………

C. **Information on poultry production**
7. What type of production do you practice?

[1] Backyard Production	[2] Free-range	[3] Intensive production

8. What types/breeds of chicken do you have?

[1] Kienyeji/local [2] Layers/ Broiler	[3] Mixed breeds / Improved Kienyeji

9. Flock size	[1] ≥10	[2] 11-50	[3] 51-100	[4] ≤101
10. Other animals kept in the farm
    (i) Poultry	[1] Yes	[2] No
    (ii) Cattle	[1] Yes	[2] No
    (iii) Pigs	[1] Yes	[2] No
    (iv) Shoats	[1] Yes	[2] No
    (v) Donkey	[1] Yes	[2] No
    (vi) Others (Specify)
D. **Information about management practices**
11. Do you allow visitors in the shed?	[1] Yes	[2] No

| 14. Have any entrance of wild animal in the farm | [1] Yes | [2] No |
| --- | --- | --- |
| 15. Source of water used in poultry? [1] Tap water | [2] Borehole | [3] Well |

[4] River	[5] Dams	[6] Others, Specify

16. How often do you clean the drinkers/water troughs? [1] Once daily [2] Once in week

[3] Twice in a week	[4] Once in a month	[5] Twice in a month

17. What type of feed do you use?

[1] Self processed/home-made	[2] Purchased/Commercial

18. How often do you clean the feeders/feed troughs [1] Once daily [2] Twice Daily

[3] Every alternate day [4] once in a month [5] Twice in a month [6] Once after production cycle

19. How often do you clean feces/floor/litter [1] Once daily	[2] Once in a week [3] Twice in a week	[4] Once in a month	[5] Twice in a month
20. Wetness and cleanliness of the enclosure **RATINGS**

| [1] = very dry [2] = moderately dry | [3] = Dry | [4] = Wet and dirty |
| --- | --- | --- |
| [5] = very wet, dirty and slippery |  |  |
| 21. Do you have any footbath in your farm? | [1] Yes | [2] No |
| 22. Do you vaccinate your birds in schedule | time? [1] Yes | [2] No |

**..........*Thank you for taking your time to fill this questionnaire..............................***
